# An innovative 3D attention mechanism for multi-label emotion classification

**DOI:** 10.1038/s41598-025-95804-2

**Published:** 2025-10-15

**Authors:** Haoran Luo, Tengfei Shao, Shenglei Li, Tomoji Kishi

**Affiliations:** Graduate School of Creative Science and Engineering, Waseda University, Tokyo, 169-8555 Japan

**Keywords:** Computer science, Information technology

## Abstract

In recent years, integrating pre-trained models with attention mechanisms has become a prevalent approach in multi-label emotion classification tasks. However, most researchers focus on modifying the attention network structure or substituting it with larger pre-trained models, often overlooking the enhancement of the attention mechanism’s learning capabilities. This paper introduces a novel attention mechanism, exemplified by the GoEmotions dataset, which encompasses 28 emotion categories, the most complex set to date. We devised distinct attention layers for each emotion label, conceptualized as separate two-dimensional planes, each containing reviews pertinent to the respective label. These planes, when stacked, form a three-dimensional cube. A multi-head attention mechanism is subsequently employed to establish connections between these planes. Additionally, we incorporated the dimensions of emotional polarity and intensity, absent in the original dataset, and defined this feature as “Commander”. The Commander functions as follows: Within each plane, it adjusts attention weight parameters among reviews via linear and non-linear transformations. Across the cube, the 28 Commanders, representing the 28 emotion labels, are determined by averaging the emotional polarity and intensity for each label. Utilizing a multi-task learning approach, we independently trained and stored predictions for each emotion. The Commanders then combine these prediction results linearly through a learnable mixing weight parameter, which is integrated into the input of the multi-head attention mechanism. We term this mechanism, which operates in a 3D attention space and guides attention learning via indicative features, as Commander Attention. When coupled with the XLNet pre-trained model for fine-tuning in downstream tasks, 3-CA outperforms the original method in the classification of all emotion categories and achieves a minimum improvement in the classification of over 85.7% of emotion categories compared to various current state-of-the-art methods. We have made the relevant core code available at https://github.com/FamerKing/Commander-Attention and will continue to update it with the complete implementation in the future.

## Introduction

Multi-label emotion classification aims to extract more detailed label information from text, images, or other modalities to categorize the content into all relevant categories correctly. This technology has excellent research significance in various applications, such as social media analysis, emotion-driven recommendation systems, and emotion-assisted interactions. The task of multi-label emotion classification deeply considers the diversity and multimodality of emotions, where a text may involve multiple emotions, and there may be mutual influences between emotions. To enable the model to recognize and classify multiple emotion labels, the use of attention mechanisms to enhance the extraction of semantic correlations and representation capabilities has gradually become mainstream.

Regarding the application of the attention mechanism in the field of Multi-label emotion classification, the following research shows considerable novelty: Yu et al.^1^ proposed a dual attention mechanism in the shared-private model enforces orthogonality between feature spaces, thereby improving multi-label emotion Classification accuracy; Huang et al.^2^ use a Hierarchical Attention-based Recurrent Neural Network, which integrates texts and the hierarchical category structure to classify documents level by level accurately; Yan et al.^3^ proposes an R-Transformer-BiLSTM model for multi-label text classification, integrating label embedding and attention mechanisms; Wei et al.^4^ introduces a multi-label text classification model with multi-level constraint augmentation and label association attention, and it addresses the challenges of label imbalance and distinguishing similar labels; GV Singh et al.^5^ introduce SENTIMOJI, a novel dataset and multi-task framework for predicting emojis in English-Hindi code-mixed text, leveraging the strong relationship between emotion, sentiment, and emojis to advance sentiment analysis and emotion recognition in code-mixed languages.

The aforementioned research has extensively explored the use of attention mechanisms. However, the datasets used in these studies have relatively limited label categories. As the number of predicted labels increases, there is a higher demand for the model’s classification capability. In 2020, Demszky et al.^6^ created the GoEmotions dataset, a large-scale dataset with 58k English Reddit reviews annotated for 27 emotion categories (including “Neutral,” totaling 28 categories). It is currently the most comprehensive and diverse publicly available dataset for multi-label emotion classification. Scholars have investigated this dataset from various perspectives. Thainguan et al.^7^ improved emotion recognition accuracy on this dataset by combining unsupervised learning algorithm LDA with popular text feature vectors. Huang et al.^8^ proposed a Seq2Emo method that implicitly models emotion correlations in a bidirectional decoder. Zanwar et al. combined Transformer models (BERT and RoBERTa) with a bidirectional long short-term memory (BiLSTM) network trained on a comprehensive set of psycholinguistic features to enhance performance on GoEmotions. Cortiz^9^ fine-tuned different Transformer language models (BERT, DistilBERT, RoBERTa, XLNet, and ELECTRA) and conducted comparisons on GoEmotions. However, all the studies above primarily focused on changing pre-trained models or considering the emotion correlations among different reviews belonging to the same emotion label. Moreover, although some domain knowledge (psychological features) was introduced, this kind of feature was not effectively utilized by the attention mechanism.

When developing our solution, we also drew some inspiration from neuroscience. The application of attention mechanisms in emotion classification tasks has been inspired by findings in neuroscience. For instance, Baldauf and Desimone^10^ demonstrated that the inferior frontal junction (IFJ) plays a critical role in selectively enhancing task-relevant visual features by modulating the neural activity of lower-level perceptual regions like the fusiform face area (FFA). This hierarchical coordination highlights the importance of modular and layered processing in efficiently recognizing and responding to emotional cues; DeVries et al.^11^, by exploring the functional specialization of different regions within the face processing network, simultaneously investigated the attentional modulation of these regions under specific frequency tagging. Through the use of neural entrainment, they demonstrated how the human brain hierarchically weights the processing of different aspects of a face across layered levels of visual processing. These studies collectively highlight the critical role of “hierarchical modules” and “attentional filters” in emotion recognition, providing new insights for designing attention mechanisms from a multi-level perspective; Poczeta et al.^12^ explore the impact of retraining reference models in multi-label text message classification systems, specifically for dynamically changing data in call/contact centers. Using Polish-language commercial data and English-language public datasets, they evaluate artificial neural networks and transformer-based models under two retraining strategies.

Therefore, this paper takes a perspective of effectively guiding the learning of the attention mechanism. It aims to explore not only the emotional fluctuations among reviews within the same emotion category but also the dependencies among different emotion labels as part of the input to the attention mechanism. Based on these considerations, this paper proposes a 3-dimensional Commander Attention (3-CA) mechanism, which makes the following key contributions:

1. We developed a 3-dimensional attention mechanism designed for multi-label emotion text. Each of the 28 emotion labels is assigned a separate attention layer, and an additional multi-head attention layer connects them. With this structural design, the emotional semantic associations among reviews within the same label and those among different labels are simultaneously considered.
2. We extend the dataset to incorporate two widely accepted emotional polarity and intensity dimensions, which we define as Commander. The Commander regulate the attention learning bias within the labels by influencing attention weights and utilizing a learnable mixing weight parameter and emotional vectors for linear blending. They are then input into the external multi-head attention to guide the learning process of emotional connections among different labels.
3. We adopt a multi-task learning approach, training the model simultaneously on multiple related tasks. This enables the model to capture shared features across tasks, extracting information from the same feature space. However, each emotion retains its own weight and biases, allowing the model to select relevant information based on the characteristics of each emotion.

## Emotional dimension expansion

In this chapter, we utilized a pre-trained RoBERTa model^13^ and the VADER emotion analyzer^14^ to predict the emotion polarity and intensity for each review in the GoEmotions dataset, thus expanding it with two new emotion features. Then, we aggregated the emotion information of all reviews associated with each emotion label by taking their mean, which became a key component of the Commander. We visually showcased the differences in the mean values of different emotion features across various emotion labels. This also indicated that there can be considerable variations in the evaluation systems for the same emotion within different contexts. We aim to utilize the structural characteristics of attention mechanisms to learn and leverage such variations as important features.

The original GoEmotions dataset only provided labels for the emotion categories corresponding to the reviews, serving as the basic logic for supervised learning. However, these labels contain limited information, and in order to provide stronger references for the attention mechanism, we needed to expand into more fine-grained and complex multidimensional emotion features. In this paper, we selected two features, emotion polarity and intensity, to enhance the initial dataset. These features will play a crucial role in the 3-CA mechanism. We chose emotion polarity and intensity as the expanded features for several reasons:

1. Polarity and intensity are fundamental aspects of emotion analysis, and prediction methods focused on these dimensions have undergone more testing and validation in the research community, making them more reliable.
2. Polarity and intensity are universal features of emotions that can be applied to text reviews across different domains and backgrounds.
3. Both polarity and intensity have high interpretability, which is beneficial for understanding model predictions and diagnosing potential issues. This is an advantage compared to other potentially more abstract or difficult-to-explain latent features.
4. Polarity and intensity are continuous features, allowing for smooth integration into attention mechanisms in a way that categorical features might not. These characteristics make them well-suited for utilization with 3-CA mechanism.

Overall, the selection of emotion polarity and intensity as expanded features offers reliability, applicability, interpretability, and compatibility with attention mechanisms, making them suitable for our intended purpose.

Emotion polarity reflects whether the emotion expressed in a text is positive, negative, or neutral. For polarity prediction, we utilize the pre-trained twitter-roberta-base-emotion model from the Transformer library, initialized with an emotion analysis pipeline. This model processes each text instance from the dataset and outputs an emotion label. The predicted labels-negative, neutral, and positive-are mapped to numerical values (− 1, 0, and 1) for computational simplicity. These polarity labels are subsequently added as new features to the enhanced dataset.

Emotion intensity quantifies the degree of emotion expressed in a text. Using the vaderEmotion library, we calculate emotion intensity scores for each text instance via its Analyzer. These scores include “compound,” “neg,” “neu,” and “pos.” The “compound” score is a normalized weighted composite ranging from − 1 (highly negative) to +1 (highly positive), derived from the “neg,” “neu,” and “pos” scores, which represent the proportions of negative, neutral, and positive content in the text (ranging from 0 to 1). For this study, the “compound” score is used as the primary measure of emotion intensity and is incorporated into the dataset as an additional feature, enabling more granular analysis of emotional content.

**Table 1:** The expanded features of reviews in GoEmotions.

Review	Label	Polarity	Neg	Neu	Pos	Compound
Thank you friend	Gratitude	1	0	0.149	0.851	0.6908
Never get out of the boat	Neutral	0	0	1	0	0
He was off by 5 minutes, not impressed	Disappointment	− 1	0.786	0.214	0	− 0.3724
Okay im interested in joining the bare hands hunting posse	Approval	1	0	0.635	0.365	0.5574
I’m going to be laughing for the next hour!	Amusement, optimism	1	0	0.696	0.304	0.5411
Wholesome and hilarious! Good job OP	Admiration, amusement	1	0	0.404	0.596	0.7088
Only $\pounds$44m? Not enough.	Disapproval	− 1	0.843	0.157	0	− 0.6914
I get all the deja smells and it’s terrible	Disgust	− 1	0.721	0.279	0	− 0.4767
Sorry, it’s just my absolute favorite thing	Admiration, remorse	1	0.14	0.538	0.322	0.4019

Table 1 shows a part of expanded features of reviews. A few observations can be made: 1. The emotion polarity feature does provide an overall emotion orientation for each review. For instance, the review “Thank you friend” which has a label of “gratitude” carries a positive emotion (Polarity: 1), reflecting the positive attitude in expressing thanks; 2. The emotion intensity value, on the other hand, quantifies the emotional strength in the text. It aligns reasonably well with the emotions conveyed in the reviews. For instance, the review “He was off by 5 minutes, not impressed” carries a negative emotion with an intensity of − 0.3724, showcasing a moderate level of negative emotion. 3. Some reviews may express more than one emotion, as seen in “I’m going to be laughing for the next hour!” which is labeled with both “amusement” and “optimism.” In such cases, the emotion polarity and intensity are still able to provide meaningful information about the overall emotional content.

These observations suggest that the additional emotion polarity and intensity features can be valuable for the multi-label emotion classification task. They not only provide another perspective for emotion representation but also offer complementary information to the emotion labels.

**Fig. 1:**
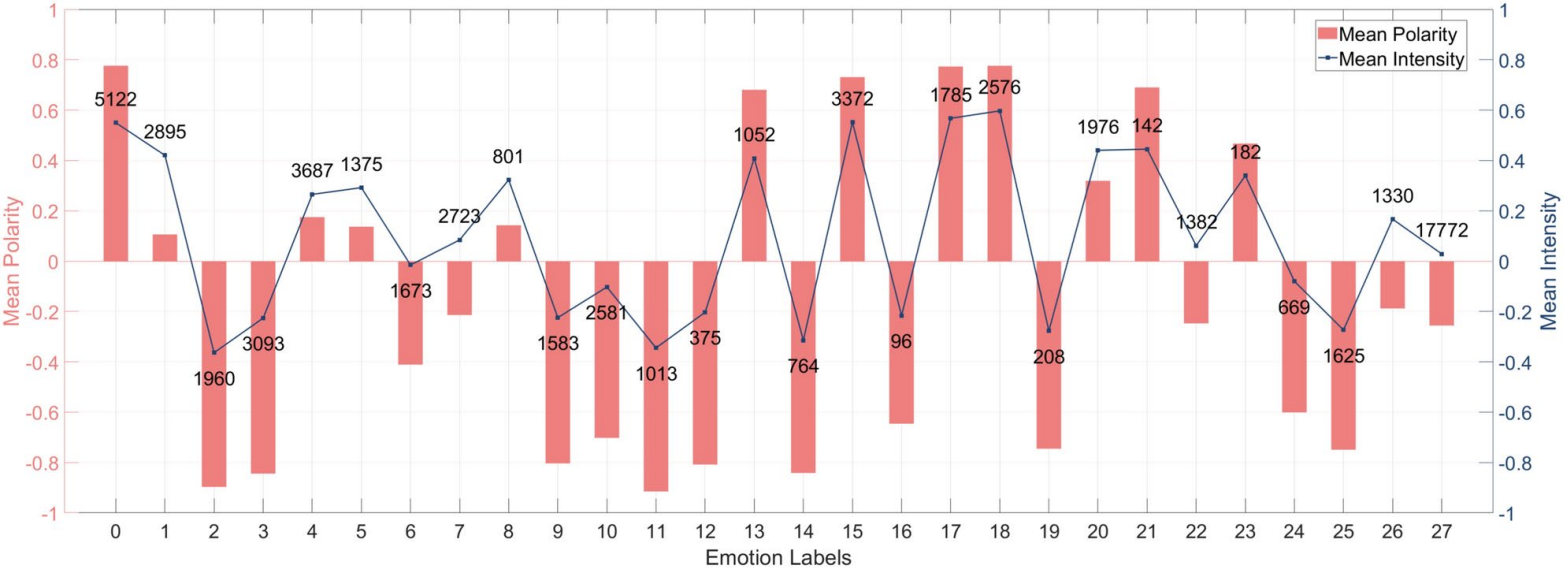
Training data after augmentation.

Next, we perform mean aggregation on the emotion polarity and intensity of reviews corresponding to the 28 emotion labels. We present the results in Fig. 1, where both sets of emotion features have the same coordinate range. Mean polarity and mean intensity are both values within the range of − 1 to 1. We use red bar charts and line charts (with node heights) to correspond to the values on the left and right axes, respectively. The line chart includes annotations above each label, indicating the number of reviews associated with it. It’s important to note that since a single review can correspond to multiple labels, the cumulative sum after separating the labels will be greater than the actual total number of reviews. From the graph, we can observe that the average emotion polarity for each label aligns with our intuitive understanding of each emotion’s polarity. Similarly, when emotions are more intense, the average intensity tends to be higher, regardless of whether it is positive or negative. On the other hand, when emotions are milder, the average emotion intensity tends to be lower.

Polarity reflects the tendency or inclination of an emotion, whereas intensity measures its strength or degree. These concepts are not directly linearly related. For instance, an emotion classified as neutral (with a polarity value close to zero) may still exhibit a non-zero intensity value (e.g., 0.028761). Neutrality does not imply the absence of emotion but rather represents a balanced state where positive and negative aspects coexist, producing an overall neutral emotion. In such cases, the intensity value captures the nuanced interplay of underlying emotions. Additionally, variations in language use when expressing neutrality may contribute to differing intensity values. Conversely, emotions with positive or negative polarity may exhibit near-zero intensity when expressed subtly or implicitly.

The average polarity or intensity values for certain emotion labels may sometimes seem unexpected, reflecting “outliers” influenced by various factors. These may include cultural differences in emotional expression, the use of figurative language, irony, or inconsistencies in dataset annotation guidelines. Such nuances underscore the complexity of human emotional expression and highlight the importance of integrating diverse emotion indicators to achieve a more holistic understanding of emotional content.

## Model construction

This chapter will introduce the model structure and construction process of the 3-CA mechanism used in this paper. We adopted a multi-task learning approach, combining the 3-CA mechanism with multiple attention structures with the upstream XLNet pre-trained model to achieve data feature extraction and multi-label emotion text classification.

### Design motivation and source of 3-CA mechanism

To better understand the motivation behind the proposed 3-CA mechanism, it is essential to first explore the origins of its design principles. The 3-CA mechanism draws inspiration from the concept of feature fusion in Natural Language Processing (NLP) but introduces innovations and improvements to adapt it to the requirements of multi-label emotion classification tasks. Specifically, 3-CA treats emotion polarity and emotion intensity as distinct features and performs feature fusion. The fused vector is then integrated with its attention mechanism, thereby enhancing its effectiveness. Specifically, the effectiveness of combining feature fusion and attention mechanisms has been demonstrated by several recent studies. Xiao et al. proposed a Multi-channel Attentive Graph Convolutional Network (MAGCN)^15^ for multimodal emotion analysis, which incorporates cross-modality interactive learning through self-attention and graph convolutional networks, as well as emotional feature fusion using multi-head self-attention. Wu et al. utilized a Multimodal Signal Fusion Network^16^ to enhance emotion analysis accuracy and explain the contributions of multimodal interactions using multi-head attention and a residual structure. Zhang et al. introduced the Integrating Consistency and Difference Networks (ICDN)^17^, which leverages attention-based cross-modal mapping and self-supervision to effectively model multimodal emotion analysis with missing modalities, achieving superior classification results on benchmark datasets. However, when applied to text-based multi-label emotion classification, these methods still face the following challenges.

**Table 2:** Normal feature fusion and the 3-CA mechanism.

Aspect	Feature fusion	3-CA mechanism
Correlation	Assumes meaningfulness	Ensures relevance
Fusion	Uses simple methods	Context-dependent blending
Domain	Universal strategy	Emotion analysis tasks
Multi-task	Does not typically involve	Incorporated
Self-attention	Secondary object	Primary object

*Challenges in existing methods*

1. Dependence on multi-modality: Most feature fusion methods rely heavily on multimodal datasets, which involve significant manual effort and cost to collect and annotate. This dependence limits the applicability of these methods to scenarios where only text data is available.
2. Over-Simplified Fusion Strategies: Many methods utilize simple fusion strategies such as concatenation, weighted summation, or stacking, which may introduce redundant information when the features being combined exhibit high correlation or dependence. For instance, in multi-label emotion classification, overlapping or interdependent emotions (e.g., “sadness” and “grief”) are common, and naive fusion approaches struggle to disentangle these relationships.
3. Neglect of Emotion-Specific Features: Existing fusion methods often fail to leverage fine-grained emotional features, such as polarity and intensity, which are critical for understanding the nuanced emotional content of text.

*Motivation for the 3-CA mechanism*

To address these challenges, this paper introduces the 3-Dimensional Commander Attention (3-CA) mechanism, specifically designed for multi-label emotion classification tasks using only textual data. The design of 3-CA draws inspiration from feature fusion methods but adapts their principles to focus on enhancing the attention mechanism’s ability to capture and utilize task-relevant emotional features. Unlike traditional fusion methods, which assume the merged features are inherently meaningful, the 3-CA mechanism explicitly incorporates emotional polarity and intensity as guiding parameters, ensuring that the fused features are both relevant and task-specific. As shown in Table 2, there are many differences between our proposed 3-CA mechanism and standard feature fusion method. Specifically, 3-CA offers the following values:

1. Task-Relevant Feature Expansion: The 3-CA mechanism introduces two key features-emotion polarity and emotion intensity-to expand the original text dataset. These features provide a more nuanced understanding of each review’s emotional content, enhancing the model’s ability to distinguish between highly correlated emotions.
2. Dynamic Fusion Approach: Instead of static concatenation or summation, 3-CA employs a dynamic fusion approach by linearly blending the averaged emotional features with the predicted outputs of each emotion category. This blended vector is passed into a multi-head attention mechanism, which captures the complex interdependencies and correlations between different emotions in a context-aware manner.
3. Independence from Multi-Modality: Unlike conventional Multimodal Sentiment Analysis (MSA) methods^18^, 3-CA focuses solely on text data, reducing the need for expensive multimodal datasets and making it more practical for real-world applications.
4. Incorporation of Multi-Task Learning: To further enhance the model’s performance, the 3-CA mechanism integrates a multi-task learning strategy, enabling the sharing of features across tasks while preserving emotion-specific weights and biases.

In summary, the 3-CA mechanism is motivated by the need to overcome the limitations of existing feature fusion methods and improve the attention mechanism’s ability to capture nuanced emotional relationships. By focusing on text-based tasks and incorporating novel emotional features, the proposed method provides a practical and effective solution for multi-label emotion classification.

### 3-CA mechanism construction

**Fig. 2:**
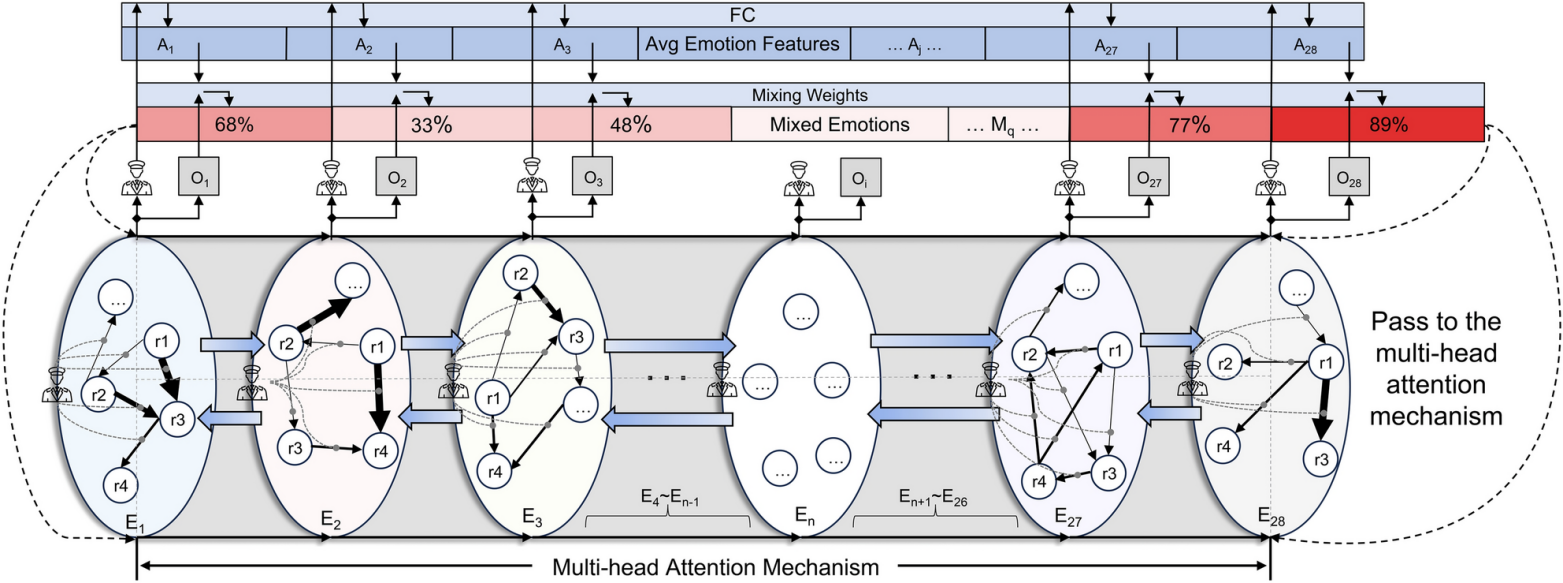
Architecture of 3-CA.

In multi-task learning, we utilize 28 independent attention layers to explore the emotional correlations among reviews corresponding to the same label. Furthermore, an additional multi-head attention mechanism is employed to establish dependency relationships among outputs of different labels. This forms the core logic of our 3-CA mechanism. Figure 2 illustrates the basic architecture of the 3-CA mechanism. Detailed explanations regarding the figure are provided below:

First, we allocate independent attention layers(planes) $E_n$ ($n \in [1, 28]$) for each emotion *e* ($e \in [1, 28]$) and its corresponding reviews. Each layer $E_n$ contains reviews r1, r2, r3, ..., rm, where m is the number of reviews associated with that label. We represent the attention layer as an ellipse. Within each layer, a Commander is depicted on the left side of the ellipse, representing the emotion polarity (*p*) and intensity (*i*) that influence the corresponding attention mechanism. The arrows between reviews indicate the degree of dependency or “attention” that one review (arrow’s starting point) has on another review (arrow’s endpoint) when forming its representation. The thickness of the arrows can be used to represent the magnitude of attention weight, where thicker arrows indicate larger attention weights. This visualization in the figure is provided as an illustrative representation. In practice, in the attention mechanism, each review considers all other reviews when generating its representation rather than just considering one-directional or pairwise relationships.

Next, we calculate the attention weights based on the Commander of each emotion. This is achieved through a series of linear transformations, activation functions, and softmax operations, as shown in Equation 1:

1 $$\begin{aligned} w_e=\operatorname {Softmax}\left( W_2 \operatorname {ReLU}\left( W_1[p, i]\right) \right) \end{aligned}$$

Here, [*p*, *i*] represents the connection of emotion polarity and intensity. $W_1$ and $W_2$ are the weight matrices of linear layers. $w_e$ represents attention weights allocated to each emotion. We compute an attention score for each token *t* in the sentence. This score is obtained by concatenating the output $O_t$ of the base Transformer model with the Commander’s input (*p* and *i*), applying a series of transformations and activations. The way of calculating the score $a_{e,t}$ is shown in Equation 2:

2 $$\begin{aligned} a_{e, t}=\operatorname {Softmax}\left( W_{5, e} \operatorname {Tanh}\left( W_{4, e}\left[ O_t, p, i\right] \right) \right) \end{aligned}$$

In this equation, $W_{4,e}$ and $W_{5,e}$ represent the weight matrices of the linear layers for each emotion *e*. After computing the attention score and emotion-specific attention weights, the Commander applies the attention weights to the output of the attention score for each token, as shown in Equation 3:

3 $$\begin{aligned} O_{e, t}^{\prime }=w_e\left( a_{e, t} O_t\right) \end{aligned}$$

Here, $O'_{e,t}$ represents the final weighted output of each token *t* under a specific emotion *e*. The Commander within the label guides the attention mechanism through the steps above to allocate appropriate attention to each review under the same emotion label. Since we employ multi-task learning, each emotion’s weighted output $O'_{e,t}$ corresponds to a fully connected layer. These outputs are then transformed into probabilities using the sigmoid activation function and finally linearly transformed to obtain the final emotions vector output. In the diagram, the emotions output is denoted as $O_i$ ($i\in [1, 28]$). Multi-task learning ensures that each emotion has its weights and biases, which helps the model select relevant information based on the characteristics of the emotion.

Then, we calculate the average emotion polarity and average emotion intensity for all reviews within each emotion label. They are computed using Equation 4:

4 $$\begin{aligned} \begin{aligned}&p_e=\frac{1}{N} \sum _{i=1}^N p_{e, i} \\&i_e=\frac{1}{N} \sum _{i=1}^N i_{e, i} \end{aligned} \end{aligned}$$

Where *N* represents the number of samples in the dataset, $p_{e,i}$ and $i_{e,i}$ represent the polarity and intensity of emotion *e* for the *i*-th sample. In the model’s input, we will include these two vectors as new features by concatenating them with the original feature vector.

Next, we calculate the average emotion features for each emotion. First, we concatenate the average emotion polarity and average emotion intensity together to form a new Commander vector of size 2. Then, we apply a fully connected(FC) layer to map them to the dimensionality of emotions. The diagram represents this step as the arrow pointing from the Commander above the ellipse to the FC layer. The output of the FC layer is the average emotion features, denoted as Equation 5:

5 $$\begin{aligned} \overrightarrow{a v g}=F C_{a v g}\left( \left[ p_e , i_e\right] \right) \end{aligned}$$

$FC_{avg}$ represents the fully connected layer used to compute the average emotion features. $[p_{e}, i_{e}]$ represents the concatenation of the average emotion polarity and average emotion intensity. During the data processing phase, since we iterate through each emotion label in a loop, the average emotion features vector elements are arranged in the order of the labels. We divide this vector into segments according to the label order, denoted as $A_j$ ($j\in [1, 28]$).

Then, we must perform a linear blending of the outputs $O_i$ and $A_j$ for each emotion. Specifically, each emotion’s output will be weighted by $w_{mix}$, and $A_j$ will be weighted by $1 - w_{mix}$. The two weighted values will be added together to obtain mixed emotions. This calculation process is defined as “Mixing Weights,” as shown in Equation 6:

6 $$\begin{aligned} O_{m i x, e, t}=w_{m i x} \cdot O_{i}+\left( 1-w_{m i x}\right) \cdot \overrightarrow{a v g} \end{aligned}$$

Where $w_{mix}$ is the mixing weight, a model parameter learned from the training data. It controls the contribution of $O_i$ and $A_j$ in the Mixed Emotions. Similar to $A_j$, the elements in $O_i$ are also arranged based on the order of emotion labels. Therefore, we operate on elements at the corresponding positions during linear blending. After this step, each emotion has an associated mixing weight, denoted as $M_q$ ($q\in [1, 28]$). $M_q$ represents the prediction for each emotion, incorporating interactions and correlations among emotions. In the diagram, the color intensity and numbers are used to indicate the probability of the model predicting the presence of a specific emotion in the text.

Finally, we apply a multi-head attention mechanism to calculate the relationships between emotions. The diagram represents this step by the dashed line extending from Mixed Emotions and pointing towards the arrows on both sides of the ellipse, indicating the multi-head attention mechanism. The continuous arrangement of arrows connects all the ellipses (attention layers), forming a 3D cylinder representing the complete composition of 3-CA.

**Fig. 3:**
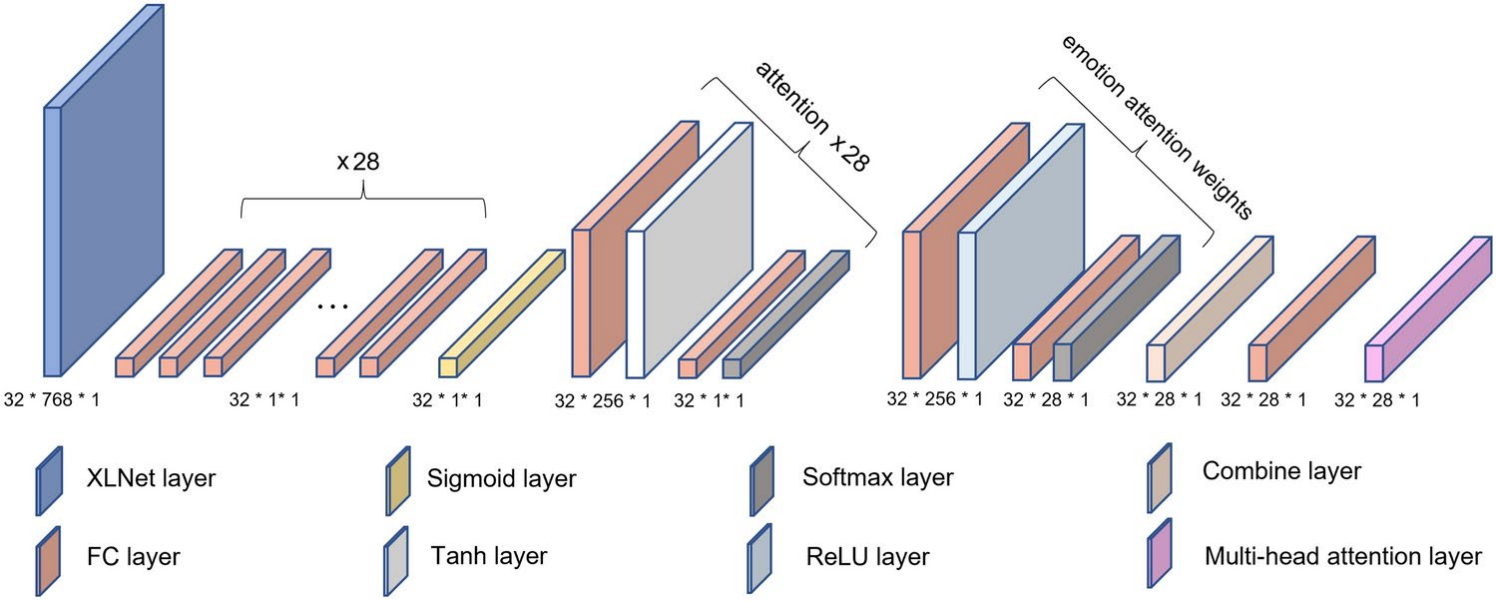
Hidden layer structure.

Figure 3 illustrates the specific hidden layer structure of the model architecture. To align with the input dimensions of the model, we introduce an additional functional dimension as the third dimension and fix its value to 1. The first two dimensions of each hidden layer represent the batch size and data length, respectively. In this structure, each attention layer primarily focuses on the importance of words for a specific emotion. The emotion attention weights layer, on the other hand, determines the relative importance of different emotions in the overall emotion prediction task. It calculates weights for each emotion based on the input polarity and intensity and uses these weights to adjust the sentence-level embeddings obtained from the attention layer. Therefore, the attention layer assigns weights to each emotion at the word level, while the emotion attention weights layer assigns weights at the emotion (label) level.

## Experiment

### Performance on GoEmotions

The experimental chapter contains the following principal contents: 1. We use different pre-trained models as baselines for comparison so as to determine the one with the best effect as a component of the feature extractor used in this paper. 2. We thoroughly test the model’s classification performance on different labels on GoEmotions, and analyze and compare it with some existing SOTA models. In order to compare with the original text of GoEmotions, we used the same Macro F1 value as the original text as the evaluation basis.

**Table 3:** Results on different pre-trained models.

Model	Precision	Recall	F1
BERT	0.45	0.51	0.48
RoBERTa	0.44	0.50	0.47
DistilBERT	0.45	0.47	0.46
ELECTRA	0.37	0.39	0.38
XLNet	0.54	0.58	0.56

**Table 4:** Comparison results across different models.

Emotions	Origin	FTLM	LLM-DT	EQN	DEFUSE	Ours
Admiration	0.65	0.73	0.68	0.71	0.69	0.72
Amusement	0.80	0.78	0.82	0.80	0.82	0.83
Anger	0.47	0.51	0.49	0.52	0.50	0.56
Annoyance	0.34	0.37	0.32	0.38	0.40	0.47
Approval	0.36	0.38	0.42	0.34	0.40	0.40
Caring	0.39	0.48	0.41	0.49	0.41	0.55
Confusion	0.37	0.44	0.44	0.45	0.42	0.43
Curiosity	0.54	0.57	0.57	0.52	0.56	0.59
Desire	0.49	0.56	0.49	0.49	0.48	0.58
Disappointment	0.28	0.32	0.26	0.34	0.30	0.36
Disapproval	0.39	0.41	0.36	0.20	0.36	0.41
Disgust	0.45	0.47	0.50	0.48	0.46	0.57
Embarrassment	0.43	0.55	0.52	0.43	0.51	0.57
Excitement	0.34	0.32	0.42	0.45	0.41	0.55
Fear	0.60	0.68	0.66	0.68	0.65	0.73
Gratitude	0.86	0.90	0.92	0.90	0.92	0.90
Grief	0.00	0.00	0.25	0.50	0.50	0.00
Joy	0.60	0.56	0.59	0.63	0.58	0.68
Love	0.78	0.78	0.79	0.81	0.78	0.82
Nervousness	0.35	0.35	0.36	0.38	0.44	0.48
Optimism	0.51	0.58	0.60	0.54	0.57	0.61
Pride	0.36	0.00	0.43	0.43	0.43	0.49
Realization	0.21	0.28	0.22	0.27	0.26	0.31
Relief	0.15	0.00	0.45	0.38	0.40	0.48
Remorse	0.66	0.77	0.72	0.60	0.65	0.66
Sadness	0.49	0.53	0.54	0.58	0.48	0.69
Surprise	0.50	0.56	0.56	0.57	0.54	0.61
Neutral	0.68	0.65	0.67	0.61	0.65	0.69
Macro-average	0.46	0.48	0.52	0.52	0.52	0.56

The experimental settings are as follows: the AdamW optimizer is used with an L2 regularization coefficient; the learning rate is set to 1e−5; the training is conducted for five epochs; and the binary cross-entropy loss function is employed. The dataset is split into training and testing sets in an 8:2 ratio. After each training epoch, the model parameters corresponding to the best performance are stored. The pre-trained models used as baselines in this paper include: 1.BERT^19^ (bert-base-cased); 2.XLNet^20^ (xlnet-base-cased); 3.RoBERTa^13^ (roberta-base-go_emotions); 4.DistilBERT^21^ (distilbert-base-uncased); 5.ELECTRA^22^ (google/electra-base-discriminator). The comparative results on the testing set are shown in Table 3.

From the table, it can be observed that the XLNet model performs the best, achieving the highest scores in all metrics. This may be attributed to its use of a larger training dataset and the Permutation-based Autoregressive language modeling approach. The differences between BERT and its variants, such as RoBERTa and DistilBERT, are relatively small, while ELECTRA performs the worst. Considering these observations, we retain the experimental results of XLNet. Next, we use our 3-CA model to compare with other SOTA methods on the GoEmotions dataset. The initial method from the GoEmotions paper is denoted as “Origin,” the previous state-of-the-art methods, fine-tuned transformers language models^9^ are labeled as “FTLM,” Large Language Models with Data Augmentation and Transfer Learning^23^ are labeled as “LLM-DT,” and the Definition-based data fusing method^24^ are labeled as “DEFUSE.” These methods, along with EQN^25^, are used as baselines for comparison with the proposed 3-CA. The results are shown in Table 4.

In terms of overall performance (macro-average), the 3-CA model (Ours) achieves a score of 0.56, showing a clear improvement compared to the Origin model (0.46) and other methods. This indicates that the 3-CA model performs better on average across all emotions and demonstrates stronger generalization capabilities. Specifically, in terms of individual emotion categories, the 3-CA model outperforms the Origin model in all 28 emotion categories (100%) in terms of macro-average scores. It surpasses FTLM in 24 categories (85.7%), LLM-DT in 24 categories (85.7%), EQN in 26 categories (92.3%), and DEFUSE in 25 categories (89.3%). The improvements are particularly notable in emotions such as “sadness,” “disgust,” and “excitement.” Additionally, for challenging emotions like “pride” and “relief,” where the FTLM model achieves a score of 0, the 3-CA model demonstrates significant improvement, indicating its enhanced robustness in handling difficult-to-recognize emotion categories. However, methods like LLM-DT, EQN, and DEFUSE manage to break the zero-recognition barrier for the “grief” emotion, outperforming 3-CA. This suggests that the 3-CA model still faces limitations when dealing with labels that have an extremely small number of samples.

### Ablation study

To evaluate the contribution of each component in the proposed 3-Dimensional Commander Attention (3-CA) mechanism, we conducted a series of ablation experiments. The goal is to systematically remove or alter different components of the model and observe the impact on its performance. All experiments were conducted under the same conditions to ensure a fair comparison. We used the same dataset (GoEmotions), training configuration, and evaluation metrics (Macro-average score, precision, and recall). The baseline model includes all components of the 3-CA mechanism: 1. Emotional polarity and intensity features (Commander); 2. Multi-head attention mechanism; 3. Multi-task learning strategy. The performance of this complete model is recorded as the baseline for comparison. We performed the following ablation experiments: A1: Remove Emotional Polarity: Remove the emotional polarity feature, retaining only the emotional intensity feature; A2: Remove Emotional Intensity: Remove the emotional intensity feature, retaining only the emotional polarity feature; A3: Remove Emotional Polarity and Intensity: Remove both emotional polarity and intensity features, using only the original emotion labels; A4: Remove Multi-Head Attention: Replace the multi-head attention mechanism with a single-head attention mechanism or remove it entirely; A5: Remove Multi-Task Learning: Remove the multi-task learning strategy, training each emotion label independently. Table 5 presents the results of the ablation experiments. For each configuration, we report the macro-F1 score, precision, and recall.

**Table 5:** Results of Ablation Experiments.

Model	Macro-average score	Precision	Recall
3-CA	0.56	0.58	0.54
A1	0.52	0.55	0.49
A2	0.53	0.54	0.52
A3	0.45	0.47	0.43
A4	0.50	0.51	0.49
A5	0.51	0.52	0.50

Overall, the emotional polarity and intensity features significantly impact the model’s performance. When emotional polarity is removed (A_1_), the macro-average score drops from 0.56 to 0.52. When emotional intensity is removed (A_2_), the macro-average score decreases to 0.53. Although the impacts of these two features are similar, the removal of emotional intensity has a slightly smaller effect on performance compared to the removal of emotional polarity, possibly because emotional intensity provides more nuanced emotional information. However, when both emotional polarity and intensity are removed simultaneously (A_3_), the macro-average score significantly drops to 0.45, indicating that these two features together contribute substantially to the model’s performance, and their absence notably reduces the model’s classification capability.

The multi-head attention mechanism (A_4_) is crucial for capturing the complex dependencies between different emotion labels. After removing the multi-head attention mechanism, the macro-average score drops to 0.50, which is a larger decline than removing either emotional polarity or intensity alone but smaller than removing both (A_3_). This suggests that the multi-head attention mechanism can partially compensate for the absence of emotional features but cannot entirely replace them.

The multi-task learning strategy (A_5_) helps the model leverage shared features across tasks, thereby improving generalization. Removing the multi-task learning strategy leads to a drop in the macro-average score to 0.51. Although the impact is smaller, it is still significant, indicating that the multi-task learning strategy plays a positive role in enhancing the model’s performance. To assess the impact of model structural adjustments on complexity and runtime efficiency, we conducted a simple evaluation. On a Tesla T4 GPU, training the dataset using the baseline method required approximately 1 hour and 30 minutes, whereas our proposed 3-CA took about 2 hours and 10 minutes. While the training efficiency is indeed slightly reduced, we believe the increase in runtime remains within an acceptable range.

**Table 6:** SemEval 2018 dataset results.

Method	F1-macro
TCS research^26^	0.530
DATN^1^	0.544
BERT-Large + DK^27^	0.563
Seq2Emo^8^	0.519
Dep-GAT^28^	0.578
RoBERTa + Modified NRC-Affect^29^	0.583
UCCA-GAT^28^	0.600
3-CA	0.616

As shown in Table 6, to evaluate the generalization performance of our model, we conducted additional experiments on the SemEval 2018 dataset^30^. The SemEval 2018 dataset contains 10,983 tweets annotated across 11 emotion categories: anger, anticipation, disgust, fear, joy, love, optimism, pessimism, sadness, surprise, and trust. Each tweet was evaluated by a minimum of seven raters, ensuring reliable annotations with high inter-rater agreement. Since 3-CA can dynamically generate the required attention planes and update the output layer to match the 11 categories, we only adjusted the number of training epochs (set to 4) and batch size (set to 16), and conducted training in the same environment. According to the experimental results, the macro-average performance of 3-CA surpassed multiple previous state-of-the-art models, achieving approximately a 3% improvement over the best-performing UCCA-GAT model.

In summary, emotional polarity and intensity features, the multi-head attention mechanism, and the multi-task learning strategy are all critical factors for the success of the 3-CA mechanism. Specifically, emotional polarity and intensity features are essential for capturing nuanced emotional variations, the multi-head attention mechanism aids in capturing complex dependencies between different emotion labels, and the multi-task learning strategy improves the model’s generalization capability.

### Interpretability analysis

In the realm of machine learning, especially in complex tasks like multi-label emotion classification, interpretability is crucial for understanding how models make predictions and ensuring their reliability. Two powerful techniques that enhance interpretability are Local Interpretable Model-agnostic Explanations (LIME) and SHapley Additive exPlanations (SHAP). LIME focuses on explaining predictions by approximating the model locally, providing insights into how slight changes in the input affect the model’s prediction for each emotion label. This helps identify which specific parts of the text influence certain emotional labels. While SHAP offers a comprehensive measure of feature importance based on cooperative game theory, giving a global perspective on how different words contribute to multiple emotion labels simultaneously. For our multi-label emotion classification task, these techniques enhance transparency, allowing us to fine-tune the model and validate its predictions by understanding the contributions of individual words and phrases to the overall emotional analysis.

To evaluate the interpretability of our model, we applied both LIME and SHAP alongside the training process. During the training, we utilized LIME and SHAP to interpret the model’s predictions. For LIME, we selected the sentence “He was just amazing, best contract ever, so sad when we lost him.” as the input. LIME perturbed this input sentence to generate a series of slightly altered versions and recorded the model’s predictions for these samples. A local surrogate model, typically a linear regression, was then fitted to these samples to explain the original prediction, highlighting the contributions of individual words to the prediction of each emotion label. Similarly, for SHAP, we used the same input sentence. SHAP values for each word in the sentence and these values were used to visualize the contributions of each word to the prediction of each emotion label.

**Fig. 4:**
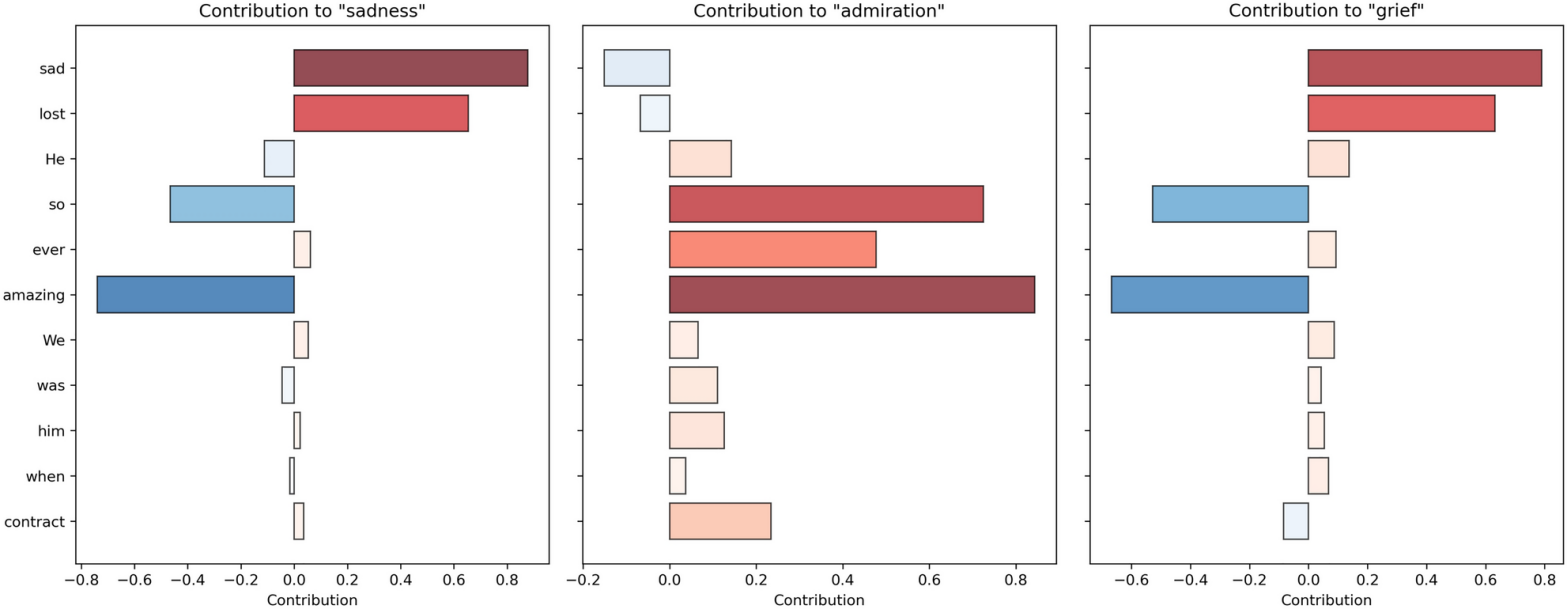
LIME result.

This sentence is associated with three emotion labels: “sadness,” “admiration,” and “grief.” Figure 4 with three subplots illustrates the contributions of individual words to these emotion labels, respectively. Each subplot’s x-axis has both positive and negative directions, representing the contribution values of each word on the y-axis to the current emotion label. Positive values indicate positive contributions, while negative values indicate negative contributions. The intensity of the color is directly proportional to the magnitude of the contribution.

In the first subplot, the words “sad” and “lost” have the highest positive contributions to the label “sadness,” indicating that these words strongly influence the model’s prediction of sadness. Conversely, words like “amazing,” “so,” and “He” have negative contributions, suggesting they reduce the likelihood of the sadness label being predicted; In the second subplot, the word “amazing” shows a significant positive contribution to the label “admiration,” which is expected given its positive connotation. The words “best” and “contract” also contribute positively, albeit to a lesser extent. Words like “He” and “ever” have a slight negative contribution, which slightly decreases the model’s prediction of admiration; In the third subplot, similar to the “sadness” label, the words “sad” and “lost” have strong positive contributions to the label “grief.” The word “amazing” also contributes positively but to a lesser extent. Words like “so” and “He” show negative contributions, indicating they diminish the likelihood of predicting grief.

Thinking further, words like “sad” and “lost” consistently have high positive contributions to both “sadness” and “grief,” reflecting their strong association with negative emotions. This consistency indicates that the model effectively captures the emotional tone conveyed by these words. The word “amazing” exhibits a strong positive contribution to “admiration” and a negative contribution to “sadness,” demonstrating its clear positive connotation. Interestingly, “amazing” also shows a positive contribution to “grief,” suggesting that the model recognizes the complex emotional context in which positive words can appear within negative sentiments. The model’s ability to identify mixed emotions within a single sentence is evident. Words contributing to both positive and negative emotions in different contexts suggest that the model can discern subtle emotional nuances, making it suitable for complex multi-label emotion classification tasks.

**Fig. 5:**
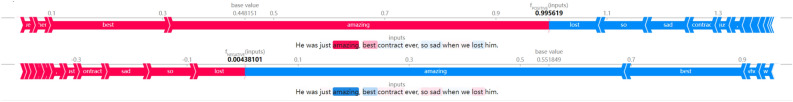
SHAP result.

Figure 5 shows the result of SHAP, taking the analysis towards to “admiration” as an example. In the positive impact section (top part of the figure), “amazing” has the highest positive contribution, significantly increasing the positive emotion score, as indicated by its large red bar. Words like “best” and “lost” also contribute positively, though to a lesser extent, as shown by their smaller red bars. “He” and “so” have minor positive contributions, which are also marked in red but are relatively small. The overall positive emotion score is about 0.996, indicating a strong positive emotional impact dominated by these words. In the negative impact section (bottom part of the figure), “lost” and “sad” have the highest negative contributions, significantly increasing the negative emotion score, as indicated by their large blue bars. Words like “contract” and “just” also contribute negatively, but their influence is less pronounced, shown by their smaller blue bars. Interestingly, words like “amazing” and “best” exhibit minor negative contributions, indicated by small blue bars, suggesting a nuanced effect where these words slightly mitigate the negative sentiment. The overall negative emotion score is about 0.00438, indicating a relatively low negative emotional impact compared to the positive score.

## Discussion

This work introduced the 3-Dimensional Commander Attention (3-CA) mechanism for multi-label emotion text classification. By incorporating emotional polarity and intensity as guiding parameters into a multi-head attention model, our approach demonstrated superior performance on the GoEmotions dataset. The Commander dimension allows for a nuanced understanding of emotions, while the multi-task learning approach captures shared characteristics across different emotion labels. These components together achieved the highest macro-F1 score in the 28-category multi-label emotion classification task, validating our method’s effectiveness.The limitations of this study mainly include the following: 1. Focus on a Single Language: The study only focuses on English, which may limit the generalizability of the 3-CA mechanism to other languages or cultural nuances; 2. Handling of Rare Categories: The model still faces challenges in handling labels with extremely few samples, highlighting its limitations in dealing with imbalanced datasets; 3. Accuracy of Emotional Polarity and Intensity Data: To obtain additional emotional dimensions absent in the original dataset, the study utilized tools like VADER for prediction. However, the accuracy of these predictions was not compared or evaluated against alternative methods, leaving room for improvement in the reliability of these features.

Future work will address the limitations identified in this study while exploring new directions to enhance the 3-CA mechanism. First, expanding the model to support multiple languages will enable an evaluation of its adaptability to linguistic and cultural diversity, thereby broadening its applicability. Second, improving the handling of imbalanced data, particularly for rare emotion categories will be a key focus, leveraging advanced techniques(such as LLMs) to address data imbalance. Third, we aim to explore alternative methods for obtaining emotional polarity and intensity values, as well as comparing various tools to enhance the reliability and accuracy of these auxiliary features. Additionally, the 3-CA mechanism could be applied to other domains such as sarcasm detection and personality analysis, while integrating additional indicators like semantic context and syntactic structure to improve adaptability. Finally, testing the model on diverse datasets across various domains and languages will help establish its robustness and effectiveness in capturing complex emotions and handling linguistic variations across cultures.

## Data Availability

The datasets and detailed models parameters used during the study are available from the corresponding author on reasonable request.
